# Mutations in genes encoding regulators of mRNA decapping and translation initiation: links to intellectual disability

**DOI:** 10.1042/BST20200109

**Published:** 2020-05-15

**Authors:** Dominique Weil, Amélie Piton, Davor Lessel, Nancy Standart

**Affiliations:** 1 Laboratoire de Biologie du Développement, Sorbonne Université, CNRS, Institut de Biologie Paris-Seine, F-75005 Paris, France; 2 Institute of Genetics and Molecular and Cellular Biology, Strasbourg University, CNRS UMR7104, INSERM U1258, 67400 Illkirch, France; 3 Institute of Human Genetics, University Medical Center Hamburg-Eppendorf, Martinistrasse 52, 20246 Hamburg, Germany; 4 Department of Biochemistry, University of Cambridge, Tennis Court Road, Cambridge CB2 1QW, U.K.

**Keywords:** intellectual disability, mRNA decay, neurodevelopmental disorders, regulation of gene expression, translation initiation

## Abstract

Intellectual disability (ID) affects at least 1% of the population, and typically presents in the first few years of life. ID is characterized by impairments in cognition and adaptive behavior and is often accompanied by further delays in language and motor skills, as seen in many neurodevelopmental disorders (NDD). Recent widespread high-throughput approaches that utilize whole-exome sequencing or whole-genome sequencing have allowed for a considerable increase in the identification of these pathogenic variants in monogenic forms of ID. Notwithstanding this progress, the molecular and cellular consequences of the identified mutations remain mostly unknown. This is particularly important as the associated protein dysfunctions are the prerequisite to the identification of targets for novel drugs of these rare disorders. Recent Next-Generation sequencing-based studies have further established that mutations in genes encoding proteins involved in RNA metabolism are a major cause of NDD. Here, we review recent studies linking germline mutations in genes encoding factors mediating mRNA decay and regulators of translation, namely DCPS, EDC3, DDX6 helicase and ID. These RNA-binding proteins have well-established roles in mRNA decapping and/or translational repression, and the mutations abrogate their ability to remove 5′ caps from mRNA, diminish their interactions with cofactors and stabilize sub-sets of transcripts. Additional genes encoding RNA helicases with roles in translation including DDX3X and DHX30 have also been linked to NDD. Given the speed in the acquisition, analysis and sharing of sequencing data, and the importance of post-transcriptional regulation for brain development, we anticipate mutations in more such factors being identified and functionally characterized.

## Introduction

Intellectual disability (ID) is characterized by significant impairments in cognitive and adaptive abilities. ID belongs to the group of neurodevelopmental disorders (NDD), which also encompasses autism spectrum disorders and many types of epilepsy among others. Genetic factors contribute significantly to NDD and a high portion of cases with ID can be explained by a single genetic event. This can be a chromosomal anomaly, a copy number variant (CNV) or a point mutation or small insertion/deletion in a single gene [1,2]. The latter represents the monogenic forms of ID, and more than 1000 genes have been identified so far, albeit each of them in only a few cases [3]. All modes of inheritance have been observed. However, in non-consanguineous populations from European ancestry, the large majority of cases are caused by a *de novo* disease-causing mutation in a gene resulting in an autosomal dominant form of ID [4], far ahead of X-linked inheritance, while autosomal recessive forms represent a residual portion of cases only (this increases largely in populations with a high consanguinity rate) [5]. The event of new sequencing technologies (high-throughput sequencing, HTS) in the last 10 years, and especially the child-parents trio whole-exome sequencing (trio-WES) approach, has allowed a considerable increase in the number of genes known to cause monogenic forms of NDD [6]. ID genes encode proteins involved in a variety of cellular processes which can be either ‘neuron specific’ such as synaptic proteins for instance but also more ubiquitous such as proteins involved in global regulation of gene expression at the transcriptional or post-transcriptional level. While the most frequent monogenic form of NDD, the fragile-X syndrome, is caused by mutations of FMRP, a ribosome-associated protein that regulates translation [7,8], the list of NDD-associated genes encoding members of RNA metabolism pathways, including those of mRNA decay and translation, is rapidly expanding [9,10]. This review will focus on a sub-set of the most recently identified ones (Table 1).

**Table 1.** Summary of genes surveyed in the review showing their associated developmental disorder, inheritance and variant types

Gene	Associated neurodevelopmental disorder (OMIM number)	Inheritance	Variant types	Ref
*DCPS*	Intellectual disability, Al-Raqad type (616459)	AR	splice and missense	[25,27]
*EDC3*	Intellectual disability, non-syndromic (616460)	AR	missense	[25]
*DDX6*	Intellectual disability, syndromic (618653)	AD	missense	[61]
*DDX3X*	Intellectual disability, syndromic (300958)	XL-D	missense or truncating	[83]
*DHX30*	Intellectual disability, syndromic (617804)	AD	missense	[91]
*DDX59*	Intellectual disability, Orofaciodigital syndrome Type V (174300)	AR	missense	[93]
*DHX16*	Intellectual disability, syndromic (618733)	AD	missense	[94]
*DHX34*	Intellectual disability, syndromic (not assigned)	AR and AD	missense or truncating	[94]
*DHX37*	Intellectual disability, syndromic (618731)	AR and AD	missense	[94]
*DDX54*	Intellectual disability, syndromic (not assigned)	AR	missense	[94]
*CNOT1*	Holoprosencephaly (618500)	AD	one recurrent missense	[95,96]
* *	Intellectual disability (not assigned)	AD	missense or truncating	[98]
*CNOT2*	Intellectual disability, syndromic (618608)	AD	intragenic deletions or truncating	[97]
*CNOT3*	Intellectual disability, syndromic (618672)	AD	missense or truncating	[99]

## Overview of mRNA decay and translation initiation

**Figure 1.**
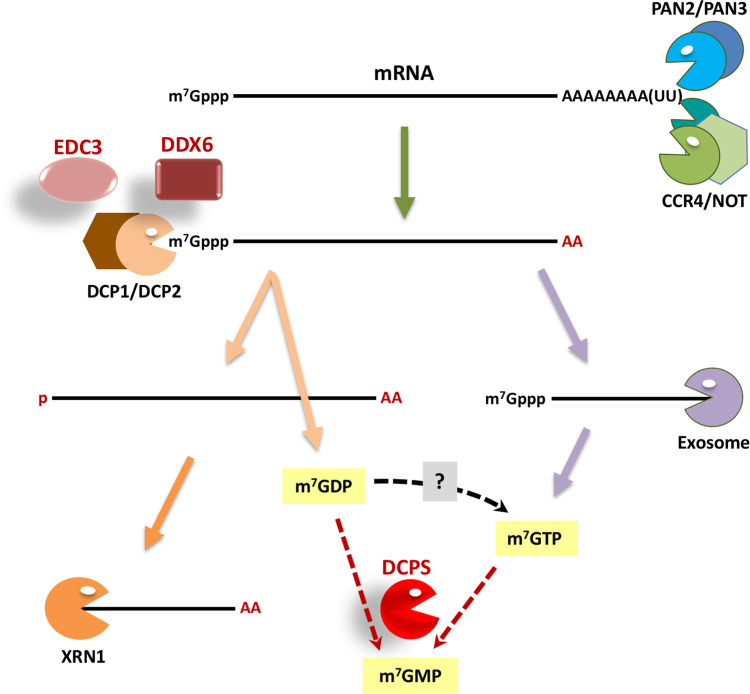
Cartoon depicting the two major mRNA decay pathways in eukaryotes.

The half-lives of eukaryotic mRNAs vary considerably, dictated by two major decay pathways in collaboration with *cis*-acting RNA elements, *trans*-acting RNA-binding proteins and microRNAs. mRNA is degraded by exonucleases which remove the 5′ and 3′ extremities of most transcripts namely the cap and the poly(A) tail (Figure 1). Decay is initiated by shortening of the 3′ poly(A) tail, catalyzed by PAN2/PAN3 and the large multisubunit CCR4-NOT deadenylase complex, and in the major decay pathway, deadenylation leads to decapping by DCP1–DCP2. This enzyme, composed of the catalytic subunit DCP2 and its essential cofactor DCP1, hydrolyzes the typical 5′ m^7^GpppG cap to m^7^GDP, which is further hydrolyzed to m^7^GMP by DCPS. Removal of the cap exposes the now monophosphorylated 5′ end of the transcript to the highly processive exonuclease XRN1, resulting in 5′-3′ decay. Several conserved activators of the decapping enzyme have been identified including the RNA-binding proteins EDC3, DDX6 and the PAT1B/LSM1–7 complex. Alternatively, deadenylation enables the RNA exosome nuclease complex access to the body of the mRNA at the 3′ end, triggering 3′-5′ decay (reviewed [11–14]).

**Figure 2.**
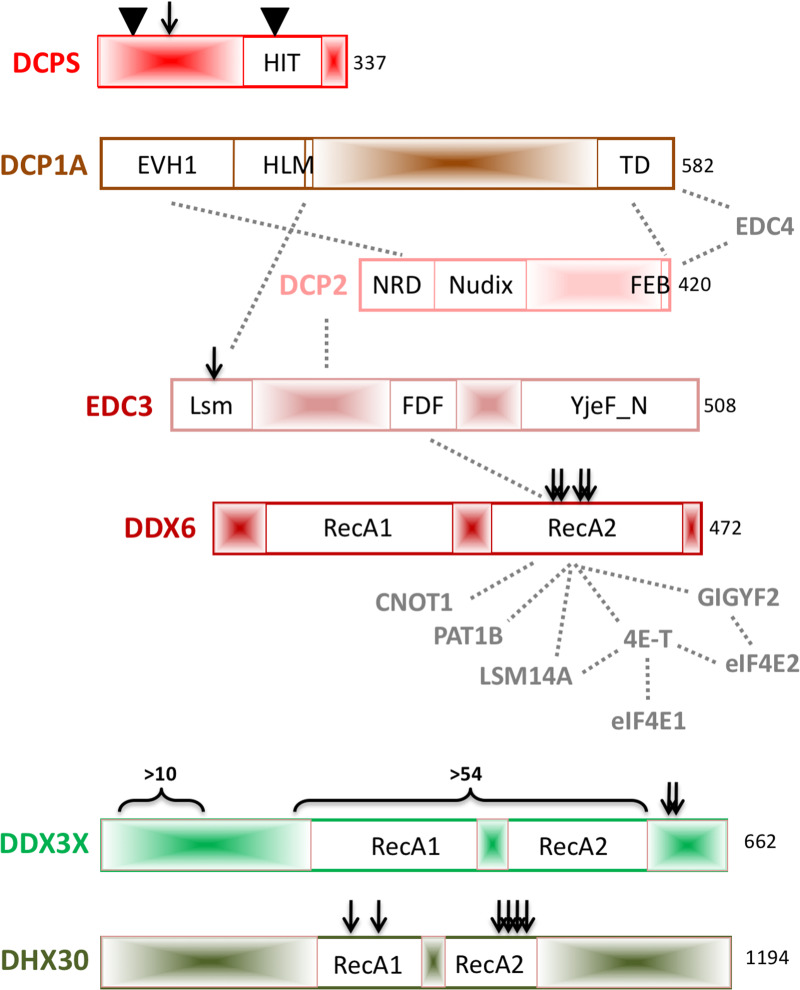
Cartoon illustrating the main domain features of human factors impacting decapping and translation initiation discussed in this review, and their interactions. Definitions not in text: DCP1A TD trimerization domain; DCP2 NRD — N-terminal conserved a-helical regulatory domain, Nudix its catalytic domain, FEB phenylalanine-rich EDC4-binding motif; EDC3 Lsm — Like Sm domain, FDF motif contains the indicated amino acids. Approximate positions of amino acid changes and insertions are indicated with blue bars and triangles. Not to scale.

The cap and poly(A) tail also play important roles in translation, by synergistically promoting the initiation step. The translation initiation factor complex eIF4F binds the cap via its eIF4E subunit and recruits the small ribosomal subunit to the 5′ end of mRNAs, while multiple PABP proteins coat the 3′ poly(A) tails. PABP interacts with eIF4G, the large multi-factor binding subunit of eIF4F, thereby enabling the formation of a closed loop of translating mRNA, in which the distal ends of a transcript circularize due to these protein–protein interactions [15]. Considerable biochemical, functional and imaging evidence supports the closed-loop model, though recent reports suggest it may operate only transiently [15,16]. Nonetheless, given the shared importance of the cap/poly(A) tail and their binding factors, the translation state of an mRNA is likely to impact its decay rate. For example, reduced eIF4E binding to the cap will not only lower translation initiation but will also destabilize mRNA. Repressors of translation include proteins that prevent eIF4E binding to the cap, such as 4E-T which sequesters the cap-binding protein and prevents its binding to eIF4G [17,18]. Interestingly, 4E-T complexes with DDX6, introduced above as an enhancer of decapping, which in turn binds the CNOT1 subunit of CCR4-NOT [19] (Figure 2), illustrating the overlap between regulators of mRNA decay and of its translation.

Regulation of gene expression at the post-transcriptional level including mRNA decay and the translation is considered at least as important as transcriptional control in many cell types [20]. This is particularly the case in the compartmentalized structure of neurons which rely extensively on regulated and localized *de novo* translation at synapses [21].

## Homozygous splice and missense variants in *DCPS* cause a syndromic form of ID

Decapping Scavenger enzyme, a conserved member of the histidine triad (HIT) pyrophosphatase superfamily, hydrolyzes m^7^GpppN to m^7^GMP and NDP. Unlike DCP2, it hydrolyzes caps even on very short RNA fragments, such as those generated by 3′-5′ exosome decay. Several reports show DCPS may also convert m^7^GDP, the product of DCP2, to m^7^GMP and Pi, but others find m^7^GDP inhibits DCPS. Regardless, m^7^GDP can convert into m^7^GTP, a substrate of DCPS, at least *in vitro* (reviewed [22,23]), suggesting that DCPS acts in both 3′-5′ and 5′-3′ mRNA decay pathways (Figure 1). As a nucleocytoplasmic shuttling protein, DCPS likely influences RNA decay in both cellular compartments [22].

DCPS enzymes are homodimers with two active sites formed in the grooves between the N- and C-terminal domains. Both domains interact with the cap and are required for catalysis. The catalytic HIT motif is located in the C-terminal domain, while the N-terminal domain binds the second nucleoside (Figure 2) [22]. DCPS activity is required to prevent the accumulation of short capped RNA fragments which may sequester cap-binding proteins such as the nuclear CBC (nuclear export) or cytoplasmic eIF4E (protein synthesis), and to reduce the possibility of stable m^7^GDP conversion to m^7^GTP which could lead to the modified nucleic acid. Homozygous deletion of *Dcps* in mice is lethal ([24] and International Mouse Phenotyping Consortium (https://www.mousephenotype.org)), as is that of *Dcp2* (IMPC).

Two studies in 2015 showed that mutations in *DCPS* result in a novel autosomal recessive disorder, manifested by syndromic ID with neuromuscular involvement, named Al-Raqad syndrome. Pathogenic mutations in *DCPS* were observed in a large Jordanian family with three affected individuals [25], and in three related patients from Pakistan [26]. In both cases, the mutations led to splice site variants resulting in short in-frame insertions (7 or 15 amino acids) between exons 1 and 2, or 4 and 5, respectively [25,26]. The levels of these *DCPS* variant transcripts [25] or encoded proteins [26] were considerably reduced in patients’ cells compared with the wild-type level in control cells, implying inefficient transcription or low mRNA/protein stability. Moreover, Ng et al. [25] noted that nuclear DCPS protein was lost in patient-derived dermal primary fibroblasts.

*In vitro* decapping tests using recombinant proteins revealed that the mutations drastically reduced the ability of DCPS to convert m^7^GpppG to m^7^GMP. Both studies also examined decapping using m^7^GpppG as substrate in patients’ cell lysates or cell lines, and here too absent or very low activity was detected, relative to unaffected family members’ or control cells [25,26]. Altogether then, the combination of low levels of mutant *DCPS* mRNA/proteins coupled with their intrinsically low decapping activity implies that the patients lack functional DCPS. Indeed, in the Pakistan family, molecular modeling suggests the 15 aa insertion caused by missplicing distorts the active site and may also interfere with the DCPS homodimer interface [26], reducing protein stability and catalytic activity. Within the family, the splice variant segregates in the homozygote state in two affected individuals while the third is a compound heterozygote with a missense variant Thr316Met. The authors showed that this missense variant also affects DCPS decapping activity. Recently, an additional individual from Italy carrying a homozygous missense variant Thr87Met was reported [27], with clinical features overlapping those previously described by Ng et al. [25]. Modeling here of the likely effects of substituting a conserved polar with a hydrophobic residue in this position suggests it could affect enzymatic activity, though such assays were not undertaken in this case study [27].

Based on overlapping clinical manifestations presented by patients from Jordan and Italy, the overall clinical presentation of the syndrome caused by bi-allelic mutations in *DCPS* consisted of severe growth delay with microcephaly, cognitive impairment, mild skeletal defects, facial dysmorphism, hypopigmentation of the skin and cardiac anomalies [27].

## A homozygous missense variant in *EDC3* as a cause of ID

EDC3 is one of the factors that enhance the activity of the major decapping enzyme DCP1-DCP2. A conserved protein, EDC3 has been particularly well characterized in fungi where it stimulates decapping *in vitro* and *in vivo* [28–32]. In contrast with the other enhancers DDX6/Dhh1, PAT1 and LSM1–7 which affect an early step in the decapping pathway, EDC3 enhances the actual cleavage reaction [28].

EDC3 proteins, members of the LSM16 family, are characterized by three globular domains, an N-terminal Lsm domain connected to an FDF domain by a low-complexity linker and a C-terminal YjeF-N-type Rossman fold domain (Figure 2). The FDF domain interacts with DDX6/Dhh1 RNA helicase [33,34], while the YjeF-N domain mediates dimerization [35], though the precise contributions of these interactions are not yet clear. In *Saccharomyces cerevisiae*, the Lsm domain binds a short linear motif (HLM) in the C-terminal domain (CTD) of Dcp2 [29,30,32], increasing the affinity of the Dcp1–Dcp2 complex for RNA substrates [13,36,37]. However, the long CTD of yeast Dcp2 is instead present in the CTD of metazoan DCP1a, as part of the so-called rewiring of the catalytic core of the decapping complex in higher eukaryotes [38]. As a result, *Drosophila* and human EDC3 use their Lsm domain to bind DCP1 HLMs [31,35,39,40], and less well documented, their linker region to bind DCP2 [35] (Figure 2).

Ahmed et al. reported a homozygous variant (Phe54Ser) located in the EDC3 Lsm domain in two affected siblings from Syria, presenting with mild non-syndromic ID. Molecular modeling predicted that the substitution of the hydrophobic phenylalanine with the polar serine disrupts the Lsm domain structure. *In vitro* assays of capped RNA and recombinant DCP2 and EDC3 proteins revealed that low concentrations of EDC3mut failed to enhance DCP2 activity, whereas high concentrations inhibited it, relative to wild-type EDC3, implying that the patients would have reduced decapping levels [26]. In light of the interactions between EDC3 and the DCP1–DCP2 complex outlined above, EDC3mut presumably affects DCP2 binding to the linker region downstream of the Lsm domain. The Lsm domain is also implicated in EDC3 cytosolic localization as it is necessary and sufficient for its enrichment in P-bodies in yeast and *Drosophila* Schneider cells [35,41]. It would thus be of interest to examine the cellular distribution of the EDC3 variant protein in patient cells or model cell lines.

In a follow-up study, Scheller et al. [42] performed a transcriptomic analysis of cells derived from these patients’ lymphocytes. In comparison with SKNBE cells, a human neuroblastoma cell line, silenced with *EDC3-*specific siRNAs, they identified ∼1–5% differentially expressed genes (DEGs). As EDC3 interacts with the AU-rich element (ARE)-binding protein tritetraspolin (TTP), which enhances the decapping of ARE-containing transcripts [43], the authors then focused on mRNAs bearing AREs. They found that ARE-containing mRNAs are preferentially stabilized in the cells with low functional EDC3 expression. More generally, long RNAs, both coding and non-coding, were up-regulated in the silenced SKNBE cells [42]. Interestingly, co-expression network analysis suggested the global association of the DEGs with synapse-related processes.

## *De novo* missense variants in *DDX6* cause a syndromic form of ID

DDX6, a highly conserved and abundant protein belongs to the DEAD-box family of RNA helicases, with two RecA-like domains containing characteristic helicase motifs including the eponymous motif II Asp-Glu-Ala-Asp, but no or short flanking regions, depending on the species.

As DDX6 proteins possess all the hallmarks of a DEAD-box helicase, including the motifs that bind either RNA or ATP, they would be expected to use ATP hydrolysis to unwind short duplexes or alter RNA–protein conformations. Nevertheless, DDX6 is a poor ATPase on its own. Structural and biochemical studies of the yeast homolog Dhh1 revealed that the two RecA domains are not flexible as in most other DEAD-box proteins, but are engaged in intramolecular interactions, restricting the ATPase activity of Dhh1 *in vitro* [34,44,45]. DDX6 proteins can bind RNA in the absence of ATP *in vitro,* unlike many helicases, and with high affinity, in the low nM range, though with little specificity [33,44–46].

In terms of function, DDX6 proteins have been characterized as enhancers of decapping and as repressors of translation, as reviewed in [47]. These roles are mediated by their interaction with DCP1–DCP2 as well as the decapping activators EDC3, PAT1B and LSM1–7 proteins on the one hand [33,34,48,49], and with translational repressor factors such as 4E-T, which sequesters the cap-binding translation initiation factor eIF4E, and LSM14, on the other [48,50–53]. Additional interactors of note include the CCR4-NOT deadenylase subunit CNOT1, whose binding enhances DDX6 ATPase activity to promote miRNA-mediated translational repression [19,51,54,55] (Figure 2). The homozygous deletion of *Ddx6* in mice is embryonic lethal (IPMC).

DDX6 proteins are found highly enriched in cytoplasmic Processing-bodies, reaching 0.5 mM concentration [46,52]. P-bodies are membrane-less RNP condensates containing specific mRNAs and RNA-binding proteins that act as storage centers of intact but inherently inefficiently translated transcripts [52,56,57]. In mammals, DDX6, in particular its ATPase activity, as well as LSM14A and 4E-T are essential for P-body assembly [48,58,59]. Moreover, the direct interactions between DDX6 and 4E-T, and between DDX6 and LSM14A, are required for efficient *de novo* P-body formation [51,60].

Balak et al. [61] identified rare heterozygous *de novo* missense variants in *DDX6* in five child patients from the unrelated U.S.A. and European families. These children presented with ID, developmental delay and similar dysmorphic features including telecanthus, epicanthus, arched eyebrows and low-set ears. Strikingly, these variants (His372Arg, Arg373Gln, Cys390Arg, Thr391Ile and Thr391Pro) are all located in a short exon encoding two conserved motifs of the second DDX6 RecA domain, QXXR and V. This exon was found to be significantly depleted of non-synonymous variation in the general population (*gnomAD*), suggesting strong selection pressure against amino acid substitutions in this region [61].

QXXR and V motifs participate in RNA-binding, ATPase activity, and protein-partner binding in DEAD-box RNA helicases, reviewed [62]. Furthermore, the second RecA domain of DDX6 has been previously shown to be sufficient for P-body localization [58]. Indeed it was observed that primary fibroblasts derived from patients with the Arg373Gln and Cys390Arg DDX6 variants have a reduced P-body number, though DDX6 protein levels were unaffected. Additional tests using model human cell lines showed that the Arg373Gln, Cys390Arg, Thr391Ile and Thr391Pro variants were capable of localizing to P-bodies, albeit inefficiently, but did not support *de novo* P-body assembly. Their interactions with 4E-T, LSM14A, PAT1B and to a lesser extent EDC3 were found to be disrupted, with Arg373Gln being the most affected. Indeed, modeling of the variants on solved protein structures showed spatial clustering of the substitutions near known interacting regions including those of 4E-T, LSM14A, PAT1B, EDC3 and CNOT1, altogether providing a reasonable explanation for their lack of higher-order condensation (Figure 2) [61].

The transcriptome of the Cys390Arg patient fibroblasts showed >1000 DEGs, relative to unrelated individuals with other neurodevelopmental or sensorineural conditions distinct from the *DDX6* mutation, with ∼500 up-regulated and ∼1000 down-regulated protein-coding genes. Similar deregulation was observed when *DDX6* was silenced in the human erythroid cell line K562 (ENCODE). Moreover, there was a significant overlap between up-regulated mRNAs and those binding DDX6, as defined using the CLIP approach (ENCODE) [61]. Many of the up-regulated genes were related to protein translation, including several *EIF3* subunit genes and about a third of ribosomal protein genes. In addition, patient up-regulated mRNAs tended to be GC-rich, as observed previously when DDX6 was depleted in model cell lines [61]. Altogether this suggests that normally DDX6 binds and silences a set of cytosolic GC-rich transcripts that is shared in different cell types and whose deregulation furthermore contributes to NDD.

The variants identified in *DDX6* adversely affect its function leading to loss of key partner binding, P-body assembly and the stabilization of some mRNAs, though the precise mechanism linking the amino acid substitutions to these outcomes is not yet clear. The variants could in principle impact the ability of DDX6 to bind RNA (in addition to reducing protein binding) or to hydrolyze ATP or to act as a helicase, or a combination of these. As P-bodies are down-regulated in patients and normally contain intact untranslated mRNAs, we speculate that *DDX6* mutations could also impact its translational repressor function [52,56,57]. Interestingly, in human stem cells depletion of DDX6 dissolves P-bodies and releases mRNAs encoding fate-instructive transcription and chromatin factors to re-enter translation, thus perturbing their self-renewal and differentiation [59]. This type of cell fate perturbation could be particularly relevant to the neurodevelopmental defect observed in patients.

## *De novo* variants in *DDX3X* are the most frequent cause of unexplained ID

The X-linked *DDX3X* gene also encodes a highly conserved and abundant member of the DEAD-box family of RNA helicases. Its two core RecA-like domains containing characteristic helicase motifs are flanked by an N-terminal nuclear export signal [63] (Figure 2). However, unlike DDX6, DDX3X is a strong RNA-dependent ATPase and helicase [64]. Structural and biochemical studies of the yeast homolog Ded1 revealed a unique auto-inhibitory interaction between the two ATPase domains in which the CTD clashes with the RNA-binding surface [65].

In terms of function, DDX3X has been implicated in various aspects of RNA metabolism from transcription, splicing to mRNA export, reviewed [63]. Notwithstanding, its major role is in the regulation of translation initiation, as first shown in yeast [66,67], though details of its mechanism of action are only beginning to emerge. DDX3X proteins interact physically and functionally with translation initiation factors including eIF4E to reduce translation or, more frequently reported, eIF3, eIF4A, eIF4G and PABP to promote translation [68–72]. A genome-wide study in yeast showed that Ded1 unwinds mRNA secondary structure in the 5′UTR during scanning of the 43S pre-initiation complex and prevents the use of near-cognate start codons that are proximal to such structures, allowing efficient translation of the main ORF [73]. DDX3X can also enhance IRES activity [74] and destabilize G-quadruplexes [75,76]. Intriguingly, recent studies propose a role of DDX3X in promoting [77] or repressing [78] repeat-associated, non-AUG (RAN) translation. Altogether, DDX3X proteins impact translation initiation, usually positively, resolving a variety of 5′UTR secondary structures, and control start codon fidelity. Homozygous deletion of *Ddx3x* in mice results in early embryonic lethality (IPMC).

DDX3X proteins are distributed in both the cytoplasm and nucleus, and have been reported to be components of neuronal RNP transport granules [79] and cytoplasmic stress granules (SG) [80,81]. SGs are large aggregates that form in response to stress and are composed of 43S pre-initiation complexes with stalled mRNAs and RNA-binding proteins [82].

Snijders Blok et al. identified truncating and missense variants in *DDX3X* as one of the most frequent causes of ID in females, accounting for ∼1–3% of all unexplained cases. Altogether, 38 females bearing 35 unique *de novo* mutations in *DDX3X* were identified by whole-exome sequencing. The mutations were either loss-of-function ones, e.g. nonsense or frameshift mutations, or missense mutations located within one of the two RecA-like domains, although rarely within one of the helicase core motifs. These girls presented with ID, developmental delay, movement disorders, behavior problems, hypotonia and epilepsy [83]. Since this initial finding, more than 100 cases bearing pathogenic mutations in the *DDX3X* gene affected by variable degrees of neurodevelopmental delay have been identified, including several male patients [84–88]. In contrast with mutations identified in *DDX6* and *DHX30* (see below), NDD-associated mutations are spread across the *DDX3X* gene (Figure 2). Yet, the Arg480 position recurrently mutated in *DDX3X* aligns with the Arg373 found mutated in *DDX6*, reinforcing the functional importance of the QXXR motif in DEAD-box proteins.

A recent study suggested that specific *DDX3X* missense mutations result in more severe clinical outcomes than loss-of-function mutations. In the developing mouse embryonic brain, the depletion of DDX3X resulted in an increased number of neuronal progenitor cells and delayed neuronal migration. *In vitro*, the missense mutations disrupted DDX3X helicase activity, and the mutation-specific reduction correlated with the severity of the disease. Furthermore, severe missense mutations decreased translation of a sub-set of mRNAs and resulted in the formation of cytoplasmic RNA-granules that accumulate puromycin, also suggestive of impaired protein translation.

## *De novo* missense mutations in *DHX30* impair global translation and cause NDD

DHX30 is a member of the DEAH-box family of RNA helicases, which mostly differs from the DEAD-box family by its DEAH motif (Figure 2). Like the two above-mentioned RNA helicases, the homozygous deletion of *Dhx30* in mice results in early embryonic lethality [89]. Regarding its function, DHX30 has so far largely escaped detailed examination, with initial studies suggesting it is involved in several phases of the RNA life cycle including ribosome assembly in mitochondria [90].

Lessel et al. identified a cohort of 12 children, carrying one of six heterozygous, *de novo*, missense mutations in the *DHX30* gene (Figure 2). All patients were affected by a severe form of NDD, presenting with global developmental delay, ID, severe speech impairment and gait abnormalities. All missense mutations affect conserved amino acids of the helicase core motifs, and most were recurrent. *In vitro* assays utilizing expression of GFP-tagged DHX30 in human HEK293T cells, confirmed that DHX30 is an RNA-dependent ATPase. Indeed, all mutations within the predicted ATP-binding motifs displayed a strongly reduced RNA-dependent ATPase activity. In contrast, no effect on ATPase activity was observed for the one mutation that lies within the RNA-binding motif Ia, and, interestingly, its phenotype was slightly distinct. To analyze the impact on RNA-binding, the authors took advantage of the publicly available CLIP dataset (ENCODE). RT-qPCR analysis of immunoprecipitated GFP-mutant DHX30 showed a reduced interaction with selected target mRNAs compared with wild-type DHX30. While DHX30 has a diffuse distribution in the cytosol, it accumulates in SGs upon heat stress. Moreover, overexpression of mutant but not wild-type DHX30 enhances SG formation, in the absence of stress, accompanied by strongly reduced protein synthesis [91]. Clearly, follow-up studies are needed to further dissect and delineate the role of DHX30 in translation.

## Concluding remarks

This review focused on the links between rare germline mutations in genes encoding a discrete set of factors that regulate mRNA decapping and translation initiation and ID/NDD. Interestingly, in the case of *DCPS*, *DDX6*, *DDX3X* and *DHX30*, their homozygous deletion in mice results in early embryonic lethality. This would imply that these factors have additional roles in embryogenesis, or that even strongly reduced functional levels enable early developmental progression. It is also of interest to consider why these mutations particularly impact brain development, though not exclusively so. Firstly, as mentioned previously, neuronal cells rely particularly extensively on regulated and localized translation compared with other cell types [21]. Secondly, the balance between proliferation and differentiation is key to brain development [92]. It is important to note that the pathology is not only in the brain, but also in a protein- and mutation-distinct manner affects face/cranium morphology, as well as other organs, such as hand/feet and heart. Moreover, the various mutations do not seem to converge on the same post-transcriptional regulation, as illustrated by the mutation in *EDC3* that enhances levels of ARE-mRNAs while they are decreased in *DDX6* mutations. Such differences presumably, at least in part, underlie the observed phenotypic variation.

Numerous further genes with a major role in various aspects of RNA metabolism have recently been linked to NDD establishing defects in mRNA metabolism as one of the major causes for this group of mostly ultra-rare diseases. To briefly mention a few such examples, Next-Generation sequencing studies in children affected by NDD/ID identified several additional underlying RNA helicase genes including *DDX59*, *DHX16*, *DHX34*, *DHX37* and *DDX54*, further implicating the role of this gene family in neuronal development and function, though the molecular processes impacted by their variants have not been characterized [93,94]. *De novo* mutations in genes encoding several subunits of the CCR4-NOT deadenylase (Figure 1) including *CNOT1*, *CNOT2* and *CNOT3* have been linked to variable NDD [95–99]. While *CNOT4* is not yet associated with any human pathology, it is highly intolerant to loss-of-function with no truncated variants in the general population, suggesting that they could have severe consequences. Whether the variant proteins impact mRNA stability and/or translation is not yet known. Mutations in the genes encoding the translation elongation eEF1 complex subunits and valyl-tRNA synthetase have also been identified as causes of NDD [100], together expanding our initial focus on mRNA decapping and translation initiation to mRNA deadenylation and translation elongation. Of particular note, a recent large-scale parent-offspring trio study of more than 30 000 affected children with developmental disorders included modeling approaches which suggested that more than 500 novel disease genes remain to be discovered [101].

Despite the identification of causal genetic factors for a growing number of NDDs for the majority of these ultra-rare monogenic disorders two main problems remain: the reliable evaluation of the pathogenicity of identified variants, and meaningful clinical interventions. It is by no means a given that every variant within a known-disease gene is a causative one. Moreover, different pathogenic (especially misssense) variants may have distinct functional effects (loss of function, gain of function, dominant negative), leading to different NDD syndromes/clinical manifestations. In many cases, the pathogenicity and thereby causality can only be proven by follow-up functional analyses. Thus, the development of reliable, time and cost-efficient functional assays is required for clinical diagnosis. Furthermore, the molecular understanding resulting from the functional characterization of underlying mutations is particularly important for the potential identification of target-based drug repurposing as a means of personalized treatment for these ultra-rare disorders.

## Perspectives

- Mutations in several genes encoding regulators of mRNA decapping and translation cause ID. While separately these variants arise very rarely, the number of such genes is increasing rapidly, reflecting progress in the use of new sequencing technologies for diagnostic purposes.
- The variants impact mRNA decapping *in vitro* (*DCPS*, *EDC3*) and *in vivo* (*EDC3*, *DDX6*), and appear to affect sub-sets of transcripts, rather than having a global effect. Much less is known of the impact of the translation regulators discussed here at the molecular level.
- Greater understanding is needed of the functional consequences of the mutations for the identification of novel drug targets for these rare disorders.

## Open Access

Open access for this article was enabled by the participation of University of Cambridge in an all-inclusive *Read & Publish* pilot with Portland Press and the Biochemical Society under a transformative agreement with JISC.

## Abbreviations

ARE: AU-rich element
CTD: C-terminal domain
DEGs: differentially expressed genes
HIT: histidine triad
ID: intellectual disability
NDD: neurodevelopmental disorders
SG: stress granules
